# On the Education of Idiots

**Published:** 1851-01-01

**Authors:** 


### ON THE EDUCATION OF IDIOTS.

Much attention has been paid of late years to this subject, and many physicians have claimed the honour of originating so important a movement. In justice to an English physician still living, we are in duty bound to call attention to his early labours in the department of psychological medicine. Dr. Richard Poole,* formerly superintendent of the Montrose Asylum, published in the year 1827, an article on "Education" in the Encyclopædia Edinensis. In this essay, under the title of "The Imbecile," he makes the following observations, on the practicability of improving the condition, and educating a large and neglected class of insane patients. Prior to the publication of these views, Dr. Poole assures us that he was not aware that any writer had anticipated his views on the education of the idiotic and imbecile. Dr. Poole is the author of the article "Mental Diseases," which appeared in the *Encyclopædia Edinensis.*

"The defects of the mind," says Dr. Poole, "or, more properly speaking, of that instrument by which the faculties of the mind are manifested, are probably as numerous, and of as frequent occurrence, as those of the animal part of our constitution. It might be possible also to subdivide them. But hitherto philosophers, with few exceptions, have contented themselves with general conclusions on the subject. It seems to have been thought enough, when any mental deficiency presented itself of a nature and magnitude which rendered ordinary education unavailing, to apply to it some such epithet as that which is placed at the head of this section; and this discovery of an incapacity for customary instruction was judged quite conclusive against the necessity of inquiry into specific differences among the unfortunate individuals who exhibited it. It is not to be wondered, therefore, that cases as dissimilar as those of the blind and the deaf, have been promiscuously comprehended under one sweeping sentence of disregard; or that any vague ideas, which philanthropy rather than science had suggested in their behalf, should prove abortive. The consequences, as might be expected, wherever the nature of a calamity, whether moral or physical, which is of frequent or extensive occurrence, is allowed to pass without suitable investigation, have devolved on our police, and that in a manner and a magnitude which positively disgrace civilized society.

"What but the most culpable ignorance, and the most culpable indifference, can account for those appalling and truly heart-rending spectacles which are so often witnessed in almost every village, and, still more marvellously, in the streets of our largest cities? Is it as a foil, one might ask, or in compliment to the usually-enjoyed proportion of intellect, that the poor idiot is permitted and encouraged, by the carelessness of his nominal keepers, in his objectless and staring perambulations among us? But if this be the motive, why is so flatteringly important a personage allowed to become the recipient of every abuse and cruelty which wantonness or fiend-like perversity thinks proper to devolve upon him? Is he not entitled, if his visitations be either profitable or tolerable, to at least the humane treatment which our laws award to the brute creation? May not even his partial and inferior resemblance to our species be somewhat enhanced by his being furnished with a decent garb, which shall protect him from the inclemencies of the weather and the harsher of insulting and prostituted superiority. Finally, is there not a possibility, if he must go at large, of guarding him against brutality and outrage, with as much care as is manifested in the preservation of property?

"In whatever manner these questions, or any similar, may be disposed of, it is certain that the evil to which they relate is of much earlier and deeper growth than the period at which such wretched beings stalk about as reproaches and nuisances to society: and the only proper efficient remedy is one, the accomplishment of which demands more profound examination, more ample command of means, and more extensive co-operation, than may at first sight be imagined necessary. Nothing could be easier, it is true, than the alleviation, if not the entire removal, of the most visibly obnoxious symptoms. The fiat of authority might compel, under severe penalties, the entire disappearance and confinement of those helpless creatures. But, admitting the efficacy and expediency of legislative interference, is it fitting for an age of improvement and

---

* Middlefield House, Aberdeen.

benevolence to allow the success of such interference, thus far, to be the ultimatum of what is desirable and practicable on the subject? Would it be, ought it to be, enough for us that those unfortunates are simply removed from our sight? We answer—No!

"It is with some anxiety, a commendable regard to decency and feeling, that we dispose of the dead bodies of our fellow-men. We protect them, too, in the last and common receptacle, by an opinion of sacredness and a rigour of law, even against the demands of an important science, which can never be duly cultivated, so as to yield its full amount of benefits, without violating a sanctuary so respected. Shall we be less concerned about the disposal of those living beings, whose weakness ought to call forth our compassion in the very proportion that it renders them burdensome to society. That there prevails a great degree of negligence as to their condition and comfort, will appear very obvious, when we compare the little attention yet shown them collectively with the extensive plans devised, at least in this country, in favour of every other class of unfortunates. Let us confine ourselves to a single city. In Edinburgh, then, we have . . . . . a great variety of other establishments for benevolent purposes. But what is done in it—what has even been attempted—in behalf of that by no means small class of helpless creatures, whom the hand of nature appears to have cast around us as if to humble our pride, and to demonstrate our dependence, for much of what we deem our excellence, on the laws of the material world? The poor-houses, it is true, usually contain some of them. But many are allowed to wander at large; and those, again, who are so lodged are, with few exceptions, precluded, by the circumstances of the establishments, and by the influence of a very general opinion, as to the total incapacity for education, from all chance or possibility of being ever useful to society. We are not certain, indeed, that there is a single institution in Great Britain exclusively, professedly, and systematically appropriated to this class of defectives.

"The reason of this neglect seems to be exactly what has been mentioned—a persuasion that there is only one species of the disease or evil under which they labour, and that this is entirely and for ever incurable. But some inquiry ought at least to be made before allowing such a conclusion; and even were this conclusion better founded than it is, there would nevertheless exist some ground for charging the practical consequences, as they are now displayed, with untenderness and impolicy, It is here contended, however, that the conclusion, in place of being warranted by facts, is disproved by them; that the mental defects of the individuals in question, so far from being all alike, are immensely dissimilar; that in many cases there is reason for imagining the principle of *substitution*, by which one faculty or sense is made to answer in some degree for another, might serve as the basis of successful education; and that it is possible the very worst cases which are ever met with would so far yield to science and industry as to vindicate and reward the patience and ingenuity bestowed on them. All that is meant to be given on the subject in this place are a few observations which, it is thought, if extended and modified by farther inquiry, might lead some benevolent minds to the adoption of a plan calculated to lessen the evil now complained of.

"Mental deficiency appears to be of two kinds—one, in which there is an imbecility or weak state of all the faculties; the other, in which there is an imperfection or a want of one faculty, or of several faculties.

"In the first, that in which all the faculties common to man exist, but in a degree inferior to that which is commonly enjoyed, there is little difficulty to be encountered in rearing the individuals to some useful occupation. Such persons are readily enough taught to a certain amount, after which they make no progress, at least no progress proportioned to the labour of instruction expended on them. An approach to this species of debility is more frequent than is generally imagined. But it is only where the case is well marked, that any departure from established treatment is required. The chief things to be attended to are the state of the bodily health and the kind of mental exercises suitable."

"There cannot, we think, be a doubt that cases of this kind, which are allowed by despair to become confirmed and deteriorated, might have been relieved by professional interference. Who has not witnessed the expressionless insane countenance, perfectly indicative of the internal state, in a person just recovering from fever, or reduced by poverty and hunger? Is it not quite conceivable that a condition of the system somewhat analogous, but dependent on causes which have operated before birth, and continued to operate even for years afterwards, might admit of an alteration and improvement similar to what occurs in these cases on the restoration of wonted health? It would not be difficult to demonstrate the truth of these remarks, and to confirm the hopes which they are intended to excite by an appeal to examples of infantine weakness followed by manual vigour. Instances are not wanting of great ability succeeding to long-continued feebleness of constitution, which did not seem to promise even mediocrity.

"In these cases, it is of the utmost consequence to proportion mental exercise to mental strength. This may be so little as to render every sort of study absolutely improper, and the very employment of the senses, beyond a certain degree, injurious. The individual must be treated at first much as a plant—and that also a sickly one—with suitable nourishment and exposure to good air. The next step is that of merely animal life, as characterized by sensations and perceptions, which will require suitable exertion. The manifestation of any of the intellectual or moral powers is an advancement of still more promising nature, and may be hailed as the basis of some moderate endeavours towards ordinary education. But throughout the whole process, great caution is necessary to guard against any overstretch of power in any direction, which would be sure to occasion a relapse, and perhaps entirely prevent recovery.

"The cases in which there occurs a defect in some one or more of the faculties are, on the whole, probably not so freqnent; but, generally speaking, they are more to be lamented. Here, however, as already hinted, there are great varieties—as the faculties themselves are numerous; and, again, the faaculty or faculties which are imperfect, or altogether wanting, may not be of great importance. It would be of no very material consequence, for example, that a person was defective in the faculty concerned in music or painting. All the concerns of life may be very well carried on without them. The same may be said of several other faculties. They are not essential to human happiness, or the common business of the world. There are instances, accordingly, of persons being destitute of them who have attained to eminence in various professions. A defect in verbal memory would be a more serious difficulty, inasmuch as it might render the individual incapable of acquiring the proper command of his mother-tongue. This is actually the chief peculiarity discernible in some idiots. The whole of their language does not, perhaps, extend beyond a dozen or two of words, and these may be often erroneously used. But the same creatures may be remarkable for some other faculty—as, for example, that on which the knowledge of places is founded, so that they may become highly useful in the capacity of guides through an intricate country which they have inhabited. On the other hand, there are instances of extraordinary verbal memory existing in individuals who were incompetent to manage the simplest affairs in life. It is quite conceivable that they might be found subservient to some useful purpose. In many idiotical persons, there is chiefly observable a total inattention to bodily wants and appearance. They have, therefore, to be reminded of the necessity of taking food, and to be forced to put on and to keep decent apparel. In some of these cases, it is not unusual to meet with singular fidelity and strength of attachment towards those who show them kindness. In others, the main peculiarity seems an entire surrender to the appetites. But even in them, unfavourable as their case is, it may be practicable to operate with some profit, as their bodily strength may often be engaged by the hope of the only reward they covet. Some idiots are noted for timidity and apprehension; others are equally so for hardihood and indifference to danger. Examples are to be met with amongst them of an unconquerable propensity to pilfer and to conceal; and occasionally one may be found possessed of an extremely ferocious disposition, and the love of mischief. It is not easy to decide, either to what good end some of these cases may be made to contribute, or in what manner they may be best restrained from doing injury. But enough, perhaps, has been said to point out the possibility of distinguishing differences in the class of defectives, and to confirm the idea that something more might be done for many, if not all of them than has usually been attempted. The philosopher—for such he would require to be—who should undertake to investigate the whole subject, and to suggest a plan of remedy or alleviation, would perform an acceptable service to science, and merit the gratitude of mankind."

It appears, from the preceding extract, that more than *twenty-three* years back, Dr. R. Poole endeavoured to awaken public and professional attention to the lamentable and neglected condition of the poor helpless idiot. Honour be to the man who, if he did not originate, certainly assisted in doing so, one of the most important and noble efforts of modern times.

